# SB Brasil 2023: Contributions from the scientific community to strengthening the National Oral Health Policy

**DOI:** 10.1590/1980-549720260034.supl.1

**Published:** 2026-10-19

**Authors:** Joana Danielle Brandão Carneiro, Marcus Cristian Muniz Conde, Érica de Sousa Ferreira, José Eudes Barroso Vieira, Ana Luiza Ferreira Rodrigues Caldas, Edson Hilan Gomes de Lucena

**Affiliations:** I Ministry of Health, Secretariat of Primary Health Care, Department of Family Health, General Coordination of Oral Health – Brasília (DF), Brazil.; II Ministry of Health, Secretariat of Primary Health Care, Department of Family Health – Brasília (DF), Brazil.; III Ministry of Health, Secretariat of Primary Health Care – Brasília (DF), Brazil.

The National Oral Health Policy (*Política Nacional de Saúde Bucal* – PNSB), known as *Brasil Sorridente*, is structured as a comprehensive public policy that coordinates a set of actions focused on health promotion, disease and health condition prevention, surveillance, and oral health care, aimed at improving living conditions and reducing oral health inequities among the Brazilian population. Within the scope of oral health surveillance, *Brasil Sorridente* encompasses different areas of action, including the monitoring of health conditions, the analysis of care-related and epidemiological indicators, and the periodic conduct of national oral health surveys, which provide essential evidence for planning and evaluating PNSB actions^1^.

One of the key remaining challenges for the *Brasil Sorridente* is consolidating a care model guided by health surveillance, capable of addressing the chronic and multifactorial nature of oral diseases while effectively integrating with other Ministry of Health policies. In this context, it is essential to establish interfaces that enable the development of an oral health surveillance model aligned with the national health surveillance policy, incorporating new approaches to measuring the impact of oral diseases on quality of life and addressing risk factors common to noncommunicable chronic diseases^2^.

This model should advance the analysis of social, economic, and behavioral determinants, strengthen information systems, and expand intersectoral partnerships to inform more equitable and effective health promotion and primary prevention strategies^3^.

For decades, Brazil has reaffirmed its commitment to the systematic production of oral health information through national population-based surveys. However, this commitment was formally institutionalized only with the enactment of Law No. 14.572/2023, whose Guideline IX established periodic national oral health surveys as a statutory requirement. This measure represents a significant regulatory advance, ensuring the continuous generation of up-to-date epidemiological data while fostering scientific and technological development in oral health^2^. This also represents an important regulatory milestone for strengthening PNSB, through the production of a primary database and care models grounded in epidemiological evidence.

Brazil has a long tradition of conducting national oral health surveys, dating to the early 1950s, within the framework of the so-called “incremental programs” implemented by the Special Public Health Service (*Serviço Especial de Saúde Pública* – Sesp), first in Aimorés, Minas Gerais (MG), and subsequently in municipalities across Brazil’s North, Northeast, and Southeast regions^4,5^. Although their scope was restricted to the school-age population, these initiatives helped to develop and consolidate important epidemiological knowledge on oral health in Brazil. However, nationwide oral health surveys only became a reality in the second half of the 1980s, beginning with the first epidemiological oral health survey in 1986, which characterized Brazil as the “toothless country.” Ten years later, in 1996, the second national survey was conducted, limited to schoolchildren in state capitals^6^. Despite their considerable importance at the time, these studies did not become established as a component of the prevailing oral health policy^5^.

Since 2000, two national oral health surveys have been conducted using representative samples of the Brazilian population, including the index ages 5 and 12 years and the age groups 15–19, 35–44, and 65–74 years, following comparable methodologies: the SB Brasil 2003 Project and the SB Brasil 2010 Oral Health Project – SB Brasil^7,8^. In addition, children aged 18–36 months were assessed in 2003. The information obtained from these surveys contributed to building a consistent oral health database for the Brazilian population, while continued nationwide surveys strengthen the institutional framework for conducting such studies^1^. The “SB Brasil 2010” oral health survey played a strategic role as an instrument of oral health surveillance within the scope of PNSB. At the time, population-based survey data revealed important progress in reducing dental caries, particularly among children and adults, and indicated expanded access to restorative dental services.

The 2023 edition of the *Saúde Bucal Brasil* Project^1^ was funded by the Ministry of Health’s General Coordination of Oral Health and conducted by Universidade Federal de Minas Gerais (UFMG), reaffirming the Ministry of Health’s commitment to building a consistent database to support informed decision-making and oral health surveillance. This partnership with UFMG underscores the importance of integration between health management and academia, which is essential for ensuring methodological rigor, scientific independence, and the training of researchers committed to improving public oral health policies in Brazil.

The national sample comprised 50,800 individuals from all regions of the country, including those aged 5 and 12 years and those in the age groups of 15–19, 35–44, and 65–74 years. Depending on the age or age group assessed, the conditions evaluated, in addition to dental caries, included the dental treatment urgency index; periodontal status; dental occlusion status; treatment urgency index; edentulism; use and need for dental prostheses; self-perception of oral health, treatment needs, the need for dental prostheses, and the impact of oral health on daily activities; care-seeking and use of oral health services; and reported oral morbidity (reports of dental pain and facial pain in the previous six months)^1^.

The results revealed both advances and persistent challenges^1^. Among 5-year-old children, 53.17% were caries-free, a positive finding that nevertheless highlights persistent inequalities, particularly given that 41.18% of the children assessed had untreated dental caries and 13.98% were already experiencing clinical consequences of the disease. Among 12-year-old adolescents, 49.88% were caries-free, whereas 36.85% had untreated lesions, reinforcing the need to develop and implement preventive strategies and expand access to oral health care. The situation was worse among adults and older adults. Adults aged 35–44 years had, on average, 10.7 teeth with a history of dental caries, and 50% had active dental caries. Among older adults aged 65–74 years, 36.48% were edentulous, and more than 70% needed dental prostheses, revealing the cumulative impact of lack of access to and discontinuity of care throughout the life course

In this context, the SB Brasil 2023 Report^1^ showed a downward trend in dental caries across all age groups evaluated. Compared with data collected in 2010, the mean dmft index among 5-year-old children decreased from 2.43 to 2.14, with the “d” (decayed) component accounting for 78.5% of the index. Among 12-year-old adolescents, the DMFT index decreased from 2.07 to 1.67, with the D component accounting for 61.85% of the index. Among adults aged 35–44 years, the DMFT index decreased from 16.8 to 10.7, with the F component accounting for 50% of the index. Among older adults aged 65–74 years, the DMFT index also decreased compared with 2010 (from 27.5 to 23.55), and the M component continued to account for the largest proportion of the index (84.3%). These results may indicate a qualitative shift in the disease profile, with fewer lost teeth and more restored teeth, suggesting greater access to appropriate therapeutic care once dental caries has become established, while also highlighting the need to address tooth loss with advancing age, with particular attention to the older adult population.

Regional inequalities persisted, with less favorable indicators in the North and Northeast regions. In these regions, dental caries in the primary dentition showed high prevalence and low treatment rates. In addition, the need for orthodontic treatment among adolescents remained significant, requiring attention to the provision and expansion of specialized oral health care, such as orthodontic care.

In a post-pandemic scenario, the findings of SB Brasil 2023 should guide the strengthening of PNSB – *Brasil Sorridente*. In this context, it is urgent and necessary to expand access, improve and innovate care, ensure water fluoridation, and reduce the inequities that characterize the oral health epidemiological profile of the Brazilian population. More than simply providing a snapshot of the population’s oral health, SB Brasil 2023 should be used as a tool for planning, evaluation, and surveillance, which are essential for consolidating a model of comprehensive and equitable care. Thus, the SB Brasil Project has established itself as a milestone in Brazilian collective oral health, and its continuity is fundamental to ensuring that the progress achieved translates into greater equity in access to oral health services, with the goal of providing quality care for the entire population.
