# Supplementary material for: SB Brasil 2023: Contributions from the scientific community to strengthening the National Oral Health Policy
Source: Rev Bras Epidemiol. 2026 Oct 19;29(Suppl 1):e260034supl1. doi: 10.1590/1980-549720260034.supl.1 (PMC13679412; doi:10.1590/1980-549720260034.supl.1)
Supplement: Translation

# Translation: SB Brasil 2023: contribuições da comunidade científica para a qualificação da Política Nacional de Saúde Bucal

**DOI:** 10.1590/1980-549720260034.supl.1.2

**Authors:** Joana Danielle Brandão Carneiro, Marcus Cristian Muniz Conde, Érica de Sousa Ferreira, José Eudes Barroso Vieira, Ana Luiza Ferreira Rodrigues Caldas, Edson Hilan Gomes de Lucena

**Affiliations:** I Ministério da Saúde, Secretaria de Atenção Primária à Saúde, Departamento de Saúde da Família, Coordenação-Geral de Saúde Bucal – Brasília (DF), Brasil.; II Ministério da Saúde, Secretaria de Atenção Primária à Saúde, Departamento de Saúde da Família – Brasília (DF), Brasil.; III Ministério da Saúde, Secretaria de Atenção Primária à Saúde – Brasília (DF), Brasil.

A Política Nacional de Saúde Bucal (PNSB) – Brasil Sorridente – se estrutura como uma política pública abrangente, que articula um conjunto de ações de promoção da saúde, prevenção de doenças e agravos, vigilância e cuidado em saúde bucal voltadas à melhoria das condições de vida e à redução das iniquidades em saúde bucal da população brasileira. No eixo da vigilância em saúde bucal, o Brasil Sorridente compreende diferentes frentes de atuação, como o monitoramento de agravos, a análise de indicadores assistenciais e epidemiológicos e a realização periódica de inquéritos nacionais de saúde bucal – fundamentais para subsidiar o planejamento e a avaliação das ações da PNSB^1^.

Um dos principais desafios que permanecem no âmbito do Brasil Sorridente é consolidar um modelo de atenção orientado pela vigilância em saúde, capaz de responder à natureza crônica e multifatorial das doenças bucais e de se integrar de forma efetiva às demais políticas públicas do Ministério da Saúde. Nesse sentido, torna-se essencial estabelecer interfaces que permitam a construção de um modelo de vigilância em saúde bucal articulado à política nacional de vigilância em saúde, incorporando novas formas de mensurar o impacto das doenças bucais na qualidade de vida e abordando os fatores de risco comuns às doenças crônicas não transmissíveis^2^.

Esse modelo deve avançar na análise dos determinantes sociais, econômicos e comportamentais, fortalecer os sistemas de informação e ampliar as parcerias intersetoriais, de modo a subsidiar estratégias de promoção da saúde e prevenção primária mais equitativas e resolutivas^3^.

Há décadas, o Brasil reafirma seu compromisso com a produção sistemática de informações em saúde bucal por meio de inquéritos nacionais de base populacional. Contudo, foi somente com a promulgação da Lei n.º 14.572/2023 que esse compromisso foi institucionalizado, ao se estabelecer formalmente, em sua diretriz IX, a obrigatoriedade da realização periódica de pesquisas nacionais de saúde bucal. Essa medida representa um avanço normativo significativo, ao assegurar a geração contínua de dados epidemiológicos atualizados e fomentar o desenvolvimento científico e tecnológico no campo da saúde bucal^2^. Isso representa um importante marco normativo para a qualificação da PNSB, com a produção de uma base de dados primários e pautada em modelos de atenção de base epidemiológica.

O Brasil tem grande tradição na realização de inquéritos nacionais, desde o início dos anos 1950, no âmbito dos denominados “programas incrementais”, implementados pelo Serviço Especial de Saúde Pública (Sesp), inicialmente em Aimorés/MG e, em seguida, em municípios do Norte, Nordeste e Sudeste^4,5^. Embora com abrangência restrita à população escolar, essas iniciativas possibilitaram desenvolver e consolidar, no país, importantes conhecimentos epidemiológicos. Contudo, inquéritos de saúde bucal de abrangência nacional se tornaram realidade apenas a partir da segunda metade dos anos 1980, com a realização do primeiro levantamento epidemiológico em saúde bucal em 1986, que apontou o Brasil como o “país dos desdentados”. Dez anos depois, foi realizado o segundo inquérito nacional, em 1996, apenas entre escolares nas capitais^6^. A despeito da grande importância que tiveram a seu tempo, esses estudos não se estabeleceram enquanto um componente da política de saúde bucal vigente^5^.

A partir do ano 2000, foram conduzidos dois levantamentos nacionais em saúde bucal, com amostra representativa da população brasileira – nas idades-índices 5 anos, 12 anos e grupos etários 15 a 19, 35 a 44 e 65 a 74 anos – e metodologias semelhantes: o Projeto SB Brasil 2003 e o Projeto Saúde Bucal Brasil 2010 – SB Brasil^7,8^. Adicionalmente, em 2003, foram também avaliadas crianças de 18 a 36 meses. As informações obtidas nesses levantamentos contribuíram para a elaboração de uma consistente base de dados em saúde bucal da população brasileira, ao passo que a continuidade de estudos de base nacional fortalece a institucionalidade dos inquéritos em saúde bucal no país^1^. O levantamento de saúde bucal “SB Brasil 2010” teve papel estratégico como instrumento da vigilância em saúde bucal no âmbito da PNSB. À época, os dados do inquérito populacional revelaram avanços importantes na redução da cárie dentária, especialmente entre crianças e adultos, e indicaram uma ampliação do acesso a serviços odontológicos restauradores.

A edição 2023 do Projeto Saúde Bucal Brasil^1^ foi financiada pela Coordenação-Geral de Saúde Bucal do Ministério da Saúde e executada pela Universidade Federal de Minas Gerais (UFMG), reafirmando o compromisso do Ministério da Saúde com a produção de uma base de dados consistente para auxiliar na tomada de decisão informada e na vigilância em saúde bucal. Essa parceria com a referida instituição de ensino evidencia a importância da integração entre a gestão e a academia, fundamental para garantir rigor metodológico, independência científica e a formação de pesquisadores comprometidos com o aprimoramento das políticas públicas de saúde bucal no país.

A amostra nacional contou com 50.800 indivíduos, abrangendo todas as regiões do país, nas idades de 5 e 12 anos e faixas etárias de 15 a 19, 35 a 44 e 65 a 74 anos. As condições avaliadas, além da cárie dentária, envolveram, a depender da idade ou faixa etária avaliada: índice de urgência de tratamento odontológico; condição periodontal; condição da oclusão dentária; índice de urgência de tratamento; edentulismo; uso e necessidade de prótese dentária; autopercepção de saúde bucal, da necessidade de tratamento, da necessidade de próteses dentárias e do impacto da saúde bucal nas atividades diárias; procura e utilização de serviços de saúde bucal; e morbidade bucal referida (relato de dor dentária e dor na face nos últimos seis meses)^1^.

Os resultados apresentados revelaram avanços e desafios persistentes^1^. Entre as crianças de 5 anos, 53,17% estavam livres de cárie, um dado que, apesar de positivo, ainda evidencia desigualdades, especialmente quando se observa que 41,18% das crianças avaliadas apresentavam cárie não tratada e 13,98% já sofriam consequências clínicas da doença. Entre adolescentes de 12 anos, 49,88% estavam livres de cárie, mas 36,85% apresentavam lesões não tratadas, o que reforça a necessidade de desenvolvimento e implementação de estratégias preventivas e de ampliação do acesso ao cuidado em saúde bucal. A situação se agravou nas faixas etárias adultas e idosas. Adultos de 35 a 44 anos apresentaram, em média, 10,7 dentes com experiência de cárie, e 50% tinham cárie ativa. Já entre os idosos de 65 a 74 anos, 36,48% eram edêntulos, e mais de 70% necessitavam de prótese dentária, revelando o impacto cumulativo da falta de acesso e da descontinuidade do cuidado ao longo da vida.

Nesse contexto, o Relatório do SB Brasil 2023^1^ apresentou uma tendência de declínio da cárie dentária em todas as faixas etárias avaliadas. Ao se realizar uma comparação com os dados coletados em 2010, a média do índice ceo-d em crianças de 5 anos caiu de 2,43 para 2,14, com o componente “c” (cariado) representando 78,5% do índice. Em adolescentes de 12 anos, o CPO-D passou de 2,07 para 1,67, com o componente C sendo responsável por 61,85% do índice. Entre adultos de 35 a 44 anos, a redução do CPO-D foi de 16,8 para 10,7, com o componente O representando 50% do índice. Na população idosa, 65 a 74 anos, o CPO-D também caiu, na comparação com 2010 (de 27,5 para 23,55), e o componente P segue representando o maior percentual deste índice (84,3%). Estes resultados podem apontar uma mudança qualitativa no perfil da doença, com menos dentes perdidos e mais dentes restaurados, indicando um maior acesso a cuidados terapêuticos adequados quando a doença cárie já se estabeleceu, ao mesmo tempo que nos mostra a necessidade de enfrentamento da perda dentária à medida que se avança na faixa etária, com especial atenção para a população idosa.

As desigualdades regionais permaneceram, com indicadores mais desfavoráveis nas regiões Norte e Nordeste. Nestas regiões, a cárie na dentição decídua apresentou alta prevalência e baixa taxa de tratamento. Ademais, a necessidade de tratamento ortodôntico em adolescentes permaneceu significativa, exigindo atenção na oferta e expansão de assistência especializada em saúde bucal, como a ortodontia.

Em um cenário pós-pandêmico, os achados do SB Brasil 2023 devem ser utilizados como orientadores para o fortalecimento da Política Nacional de Saúde Bucal – Brasil Sorridente. Nesse contexto, mostra-se urgente e necessário ampliar o acesso, qualificar e inovar a atenção, garantir a fluoretação das águas e reduzir as iniquidades que marcam o perfil epidemiológico de saúde bucal da população brasileira. Mais do que um retrato da saúde bucal da população, o SB Brasil 2023 deverá ser utilizado como um instrumento de planejamento, avaliação e vigilância, essencial para a consolidação de um modelo de atenção integral e equitativo. Assim, o Projeto SB Brasil se consolida como um marco da saúde bucal coletiva brasileira e sua continuidade é fundamental para garantir que os avanços conquistados se traduzam em promoção da equidade no acesso aos serviços de saúde bucal, objetivando o cuidado qualificado para toda a população.
